# Applying lessons learned from anthrax case history to other scenarios.

**DOI:** 10.3201/eid0504.990420

**Published:** 1999

**Authors:** J G Bartlett

**Affiliations:** Johns Hopkins School of Medicine, Baltimore, Maryland, USA. biodefen@jhsph.edu


### Applying Lessons Learned from Anthrax Case History To Other Scenarios

**John G. Bartlett**
Johns Hopkins School of Medicine, Baltimore, Maryland, USA

Northeast, the city described in the anthrax scenario (Inglesby, this issue, pp. 556-60) is actually Baltimore, a metropolitan area of 2 million population, with a football stadium that holds 74,000. Route 95 would be where the anthrax dispersion took place.

My test case started on February 13 at 6 a.m. when I went to the emergency room at Johns Hopkins University Hospital and asked to see the physician in charge. I described the typical case and asked what the procedure would be if a patient came down with these symptoms. The physician in charge had actually taken the specialized 8-hour training course on bioterrorism (one of five physicians in Maryland to have completed this course entitled "Train the Trainer"). Nevertheless, she confessed that the typical early case of inhalation anthrax would have a presumed diagnosis of flu, and the patient would probably be sent home. Despite the emphasis on emergency room physicians as the "early response team," the actual diagnosis would be made after hospitalization. Many seriously ill patients arriving at the same time might arouse suspicion, but the initial cases would likely be isolated events or would be dispersed in multiple emergency rooms.

There was a further problem. At the time of my visit, the emergency room was on "blue alert," meaning that all 28 beds were filled; the hospital was also filled. Furthermore, the whole city was on blue alert, probably because of the flu epidemic. Hospitals routinely run on marginal excess capacity. The pressures of managed care have resulted in a health-care system that has minimal elasticity, so on February 13, there were no beds for an anthrax epidemic.

Address for correspondence: John G. Bartlett, Johns Hopkins Center for Civilian Biodefense Studies, Candler Building, Suite 850, 111 Market Place, Baltimore, MD 21202, USA; fax: 410-223-1665; e-mail: biodefen@jhsph.edu.

I then went to radiology; I showed the radiologist a classic case of inhalation anthrax and asked him how he would interpret the X-ray. He said that he would read it as widened mediastinum; the differential diagnosis did not include anthrax.

Then I went to the laboratory and asked the lead technician who has been in the laboratory for 25 years. He said that *Bacillus anthraxis* had never been isolated during his tenure. If it was recovered in blood cultures, it would be called "*Bacillus* species, a probable contaminant." However, more than three cases of *Bacillus* species would prompt a full identification, which would be available in 48 hours. That would trigger a call to the chief of Infectious Disease and to Infection Control. It would take 72 hours to get sensitivity test results—which is important since this information would drive the subsequent decisions regarding antibiotic prophylaxis to those patients or persons who had been exposed. My own response (if given the possibility of a case of inhalation anthrax) would be to call the state health department—the Maryland Department of Health and Mental Hygiene.

I got a recording and left a message that I had a query about bioterrorism, and it was important. The call was returned 3 days later. The state does have a response mechanism that is far along in planning and can be activated with a single phone call. The problem is that I did not know the number. No one else seemed to know the number; it is not in the hospital directory or on 911 listings.

How were we set in Baltimore to deal with antibiotics? What was the supply? At any moment, the city of Baltimore had 69,000 capsules of ciprofloxicin and 99,000 capsules of doxycycline. We could probably use a number of other flouroquinolones, and if the sensitivities proved that penicillin was active, we could use that as well. Access to antibiotics would not be a major problem in this scenario of anthrax contamination.

Then I reviewed the statewide facilities and planning for a bioterrorist attack. One phone call to the state health department would set into motion a cascade of events that would include an immediate effort by state epidemiologists to review the data and confirm the diagnosis. They would then contact the Maryland Emergency Management Agency, the Federal Bureau of Investigation, Maryland Institute for Emergency Medical Services System, and other appropriate agencies. The Maryland Emergency Medical Agency coordinates relevant state agencies and also acts as spokesperson to the press.

Maryland Institute for Emergency Medical Services System has the capability for flash faxes to emergency rooms throughout the state but does not communicate with infection control programs and other parts of the hospital because somebody in the emergency room can always get that information. My perception is that Maryland does not have a good system to reach its practicing physicians, whose involvement is critical. To give antibiotics to tens or hundreds of thousands of persons in several days, it will be necessary to use more than the health department clinics and personnel. Notification and direction would have to be done through the press and through the medical society, but it is not clear how well this would work. There had been a few examples, however, of how this system would work in other settings. The Maryland Emergency Medical Agency, the system for public communication, is active about two to three times a year, primarily for ice storms and hurricanes. It has not been tested for a major epidemic, but at least it is a system that is established. The capacity for bodies in a morgue would be approximately 100, but there are contracts to get refrigerated trucks that would hold 40 bodies per truck. The system is set up so that Maryland Institute for Emergency Medical Services System can readily identify bed capacity for every hospital in Maryland including the number of available intensive care unit beds to facilitate referrals. No plan is available for stockpiling antibiotics or vaccines. Stockpiling of antibiotics is not necessary because the city could get an adequate supply from regional sites, and the Centers for Disease Control and Prevention has a $50 million budget allocated to this need. The great need is for deploying antibiotics in an expeditious way to thousands, presumably by using regional care sites and the thousands of physicians' offices; 3,000 emergency medical service providers could be available to assist, but the mainstay of care in any large epidemic would come from the private sector.

How does all this work? The good news is that we have a system set up where there is one person or one group that is coordinating the events and one point of contact that initiates the relevant cascade of events necessary for a response. Can this system respond the way it is expected to respond? The system has worked in natural disasters, but it may break down in a large outbreak of inhalation anthrax. For example, during a pfisteria crisis, many groups took the outbreak on as their issue. Representatives of Congress and influential citizens bypassed the governor, the mayor, the Maryland Emergency Management Agency and every other system to contact the White House, CDC, other agencies and various medical experts to deal with it. Many did not like the answers they got, so they bypassed standard channels, and many are unaware of the rules. A system with a single voice for communication with the press and providers is needed. The state has 13,000 beds, but a flu epidemic recently overwhelmed hospital capacity, and this was not even a big year for influenza. A recent large fire in Baltimore demonstrated that the city could not handle 100 casualties.

Finally, there is the issue of medical-care personnel resources to respond. Maryland has 16,000 physicians, 262 members of the Infectious Disease Society, and 400 emergency room physicians; in addition, every hospital has infection control personnel. In the event of a bioterrorist attack, these will be the first responders. They are the front line for patient contact with the health system. They will suspect or establish the diagnosis, develop systems to regulate hospital flow, make therapeutic policy, give treatment, and will provide prophylactic antibiotics and vaccines. Federal, state, and local health agencies play a central role in planning but do not have the facilities or field forces necessary to deal with sick patients and the thousands who need vaccines or antibiotics.

The gap in planning at the federal level has been the failure to include these diverse groups at the table. We anticipate two responses. Different groups will make territorial claims on the issue; infectious disease physicians will say bioterrorism is what they are trained for, infection control practitioners will claim that epidemics are their special skill, emergency room physicians will claim that they will be the first to see those patients, and microbiologists will claim that they make the diagnosis. All have a role, and all should be included. The second response seems diametrically opposed. We suspect that it will be difficult to engender participation by relevant groups, despite their claims regarding discipline relevance. A bioterrorist attack is a low-probability event for nearly all cities when considered individually. Cleveland, Tulsa, or Sacramento are unlikely targets, just as Oklahoma City was an unlikely target. Medical providers are busy, and most of us have volunteered to the breaking point. It is not surprising that the "Train the Trainers" sessions on bioterrorism in Baltimore were attended by only five emergency room physicians and no representative of hospitals. Thus, enthusiastic participation by the critical players from the private sector is unlikely.

The major mechanism for recruitment is a carrot or a stick. Possibilities include making bioterrorism plans by hospitals a Joint Commission on Accreditation of Organization requirement, requiring this in RRC selected training programs, asking it on American Board of Internal Medicine boards, and incorporating it in medical school curricula. These possibilities would increase visibility of the issue but would not provide the proper regional training needed. The resources that now total $20 million should include an allocation to the private sector to permit training and planning programs that represent a true partnership between public and private sources.

Dr. Bartlett is professor and chief of the Division of Infectious Diseases, Johns Hopkins University School of Medicine and president of the Infectious Diseases Society of America.

